# Outwitting dengue threat and epidemics resurgence in Asia-Pacific countries: strengthening integrated dengue surveillance, monitoring and response systems

**DOI:** 10.1186/s40249-016-0148-3

**Published:** 2016-05-27

**Authors:** Ernest Tambo, Jun-Hu Chen, Xiao-Nong Zhou, Emad I. M. Khater

**Affiliations:** Department of Biochemistry and Pharmaceutical Sciences, Higher Institute of Public Health Sciences, Université des Montagnes, Bangangté, Cameroon; Africa Disease Intelligence and Surveillance, Communication and Response (Africa DISCoR) Foundation, Yaoundé, Cameroon; National Institute of Parasitic Diseases, Chinese Center for Disease Control and Prevention, Shanghai, 200025 People’s Republic of China; Key Laboratory of Parasite and Vector Biology of the Chinese Ministry of Health, Shanghai, 200025 People’s Republic of China; WHO Collaborating Centre for Tropical Diseases, Shanghai, 200025 People’s Republic of China; Public Health Pests Laboratory, Jeddah, Jeddah Governate Saudi Arabia

**Keywords:** Dengue, Epidemics, Resurgence, Surveillance, Monitoring, Response, China, Asia-Pacific

## Abstract

Dengue is still a substantial vector-borne viral disease threat and burden of public health importance worldwide. This situation is complicated by dengue virus unprecedented resurgence and persistence of varied serotypes in endemic-prone areas, and man-made and natural activities consequences that promote vector emergence, transmission dynamics and spread across the Asia-Pacific region. There is an urgent need to strengthen operational and contextual surveillance-response research in improving early detection of active reservoir detection, novel drug in case management and quality evidence-based response including the deployment of dengue mass vaccination. Moreover, sustained mapping and watching of dengue risk factors or determinants, performance and outcome indicators of control or elimination programs effectiveness in defining minimum effective data towards community knowledge-based decision-making policy and effective response packages is imperative. Moreover, implementation of a robust, integrated dengue early warning surveillance, monitoring and response systems metrics is required for evidence-based, timely and cost-effective contextual mitigation strategies, and innovative interventions.

**Electronic supplementary material:**

The online version of this article (doi:10.1186/s40249-016-0148-3) contains supplementary material, which is available to authorized users.

## Multilingual abstracts

Please see Additional file 1 for translations of the abstract into the six official working languages of the United Nations.

## Background

The changing dengue socio-ecological and epidemiology resurgence in Asia-Pacific countries represents a vector-borne viral disease of increasing public health importance. The increasing dengue threat and epidemics resurgence to local populations and international community with intense urbanization and the growing global travel and trade is worrisome [1]. Since 1955, more than 100 countries with a total of 284–528 million dengue cases, of which 67–528 million cases showing symptoms and 82 000 deaths according to the World Health Organization (WHO). Hemorrhagic dengue fever (DHF) and dengue fever (DF) can be fatal, accompanied with fluid accumulation, plasma leaking, respiratory distress, severe bleeding and organ impairment. About 500,000 are estimated to contract dengue long-term complications that require hospitalization, leading to about 22 000 deaths each year, according to the WHO report. The availability of data on DengueNet has declined dramatically after 2005 through the national dengue control programs [2]. South-East Asia and Western Pacific regions are the most affected areas with more than 1.2 million cases in 2010, with an increasing trend in 70–75 % in risky populations in Indonesia, Thailand, Cambodia, VietNam, Laos PRD, Myanmar, the Philippines, Singapore, Malaysia, Cook Islands, Fiji, Vanuatu and outbreaks in India, Sri Lanka and the Maldives in recent years. In China, type 4 DHF epidemic have occurred in Shiwan town, Foshan city, Guangdong province, southern China in 1978 with 583 hospitalized patients. Later occurrence of type 1 was in Shiqi town, Zhongshan County, Guangdong province in 1979 and the largest type 3 documented on Hainan Island in 1980 with 510 hospitalized patients and four deaths [2].

*Aedes *reemergence has also be documented in Hainan island in 1985 and 1986 occurred across aged groups with major symptoms ranging from acute intravascular haemolysis and multiple peripheral paralysis, diffuse intravascular coagulation, loss of hair and altered mental status which might likely compound the burden of emerging and persisting vector-borne diseases [1–3].

Achieving the global dengue strategy, which calls for at least 50 % reducing the disease mortality burden and a minimum of a 25 % reduction in incidence by 2020, requires more governmental, private sector and other stakeholders commitment, investment and reliance on innovative approaches and interventions at all levels. Nonetheless, various dengue national control programs in Southeastern Asia, Western Pacific, Africa, South-Eastern America and the Caribbean regions haven’t sufficiently fostered financial investment needed in understanding comprehensively knowledge gaps and in providing long-lasting protection/solutions to vulnerable populations needs. In such effective and safe dengue drugs and vaccines development and mass deployment are urgently needed to support Asia-Pacific Strategy for Emerging Diseases goals declarations of 2005 and 2010 and novel cross-border platform for dengue data and information sharing, and joint collaborative research projects on vector-borne diseases of public health importance [1, 2].

In response to the recent rising global dengue burden, China dengue biological control approach led to the inbred mosquitoes engineering of more than half a million male mosquitoes at science park laboratory, Chinese Center for Disease Control and Prevention and the Guangzhou Center for Disease Control and Prevention in Guangdong province are being released at Shazai, a 3-sq-km island in a Guangzhou suburb since March 12, 2015. These inbred mosquitoes survive for about two weeks, and their reproduction capacity reaches its peak within the first few days of release. They also have potential to make their female mating partners infertile on an island in Southern China every week on the fight against dengue fever and novel approach in other vector borne diseases management [4, 5]. Last year, China reported about 46,000 dengue fever cases and five deaths compared with around 1,000 in the previous years, mostly in Guangdong province. Also eggs produced by mosquitoes that mated with *Wolbachia*-infected male counterparts are infertile [4–6]. This biological application in mass population dengue prevention in most endemic areas has been proven effective in *Aedes* mosquito population reduction, but requires further clinical trials and long - term pharmacovigilance.

The new threat and Zika viral epidemic that continue to rag the Caribbean regions and part of the Americas (South and Central), has prompted WHO to declare global health emergency. Strengthening global coalitions against dengue, Zika and related arboviral diseases (Chikungunya, dengue, Rift Valley) underscore the potential impacts of ZIKV on maternal-child health and long-term Zika consequences on survivors with documented new born babies with microcephaly, Guillain-Baurre syndrome and other neurological complications to stillbirth [7].

The erratic and inconsistency of Zika and dengue vectors (*Aedes aegypti, A. albopictus*) surveillance and lack of key performance indicators measurements of vector control programs in most endemic countries require urgent attention, development and implementation based on robust contextual evidence translation, technical and non-technical support systems. Sustained monitoring and evaluation systems are needed for effective and continuous *Aedes* mapping and threat communication on differential local/national and regional integrated vector management programs. The absence of coordinated national and regional Zika and other vector-borne diseases and lack immunization programs severely hampered that coordinated programmatic and evidence-based prevention and control approaches and interventions over space and time [6, 7]. Notwithstanding, the existing national dengue surveillance sentinel sites, the persistent resurgence of dengue incidence and prevalence is been documented in townships in Pearl River Delta and Yunnan in 2009, 2013 and 2014, respectively [6–8]. Hence, the call to invest in strengthening more robust surveillance and monitoring systems (including resistance) is essential in providing critical evidence-based knowledge and information in informed-policies towards well-timed effective local mainland and regional programmes. It is essential to tackle firmly *Aedes* vector expansion into new areas and new threat in non-immune populations, Zika and dengue epidemics changing dynamics health and economic consequences worldwide [7, 9]. Other critical factors that require paramount mitigation actions and measures include climate changes, globalization of travel and trade and intense urbanization and increasing population density impacts those offer more favorable advantages to new *Aedes* mosquito ecological development suitability and spread [6, 7].

Increasingly evidence-based public health priorities and actions have been recommended and implemented based on a wide range of disease surveillance systems and minimum effective data generation. Strengthening public health pests laboratory is essential in establishing and sustaining integrated pest and disease surveillance-response information and knowledge generation in advocacy and coherent and effective response programs in threats, and epidemics in vulnerable countries [9, 10]. However, it is necessary to develop and integrate a comprehensive innovative national and regional quality and coherent surveillance data and key performance indicators, which is necessary in guiding sustainable health planning, effective control and prevention of disease threat and distribution. Although, health programming and economic development have been challenged by a number of factors, the increasing use of surveillance and monitoring systems as critical cornerstone in improving preventive and control measures and timely emergency response in epidemics has been advocated to support evidence-based threat mitigation and averting potential dengue epidemics [10, 11]. Such effective local and cross borders migration/travel information data should be gathered and analyzed in understanding the fundamental epidemiological, ecological, climatic and socio-behavioural factors that can enhance dengue resurgence and epidemics forecasting and timely collaborative actions [9, 11–13].

First, leadership commitment and financial investment efforts of China and other Asia-Pacific governments and stakeholders from various levels and long-term collaborative mechanism along with community and private sectors should be established in *Aedes *mosquito and dengue sustained control and elimination priorities. Operational research is needed in generating reliable, cost-effective and practical evidence for innovative decision policy and approaches to outwit dengue and other emerging infectious diseases reservoirs, understanding dengue vector competence determinants and disease etiology, improving more sensitivity and field adaptable rapid diagnostic techniques. Accelerating dengue drugs and vaccines discovery in transmission blocking is crucial in filling knowledge gaps, implementation of comprehensive and practical control policies and solutions [2, 8, 10]. Especially, increasing advocacy and social mobilization on the importance of integrated laboratory surveillance and monitoring system in scaling up data and information access and sharing is paramount in increasing faith-based and community-led capacity development, targeted awareness, health education and vigilance. This requires commitment and funding from local private sector, international funding agencies and regional organizations, pharmaceutical companies, academia, non-governmental and the governments in curbing the increasing global *Aedes* spread [14].

Second, the importance of knowledge-driven innovations in integrated vector management (IVM) methods and tools cannot be overemphasized in dengue and related vectors transmission interruption. Thus, establishing of key performance indicators (KPIs) is vital in measuring existing and current surveillance and vector programs effectiveness, cost effectiveness and achievements. In such assessing reductions in vector population density and vector longevity or vectorial capacity including entomological inoculation rate (EIR), *Aedes* house and population density indices, human biting rate, container breeding index, the Breteau index and community KAP index, Aedes susceptibility/resistance index, adult mosquito density and competence are needed. Moreover, understanding dengue vector distribution, behaviour changes in risky populations, cultural ecology and environment context should be conducted towards coherent implementation of appropriate evidence-based, integrated and sustained health, agriculture and forestry to public health veterinary management programs and action plans. Coordinated and directed community participation in self-protection consciousness and resilience in prevention among risky populations and global community is necessary to compress the endemicity and potential future emergency outbreaks. Applications of laboratory proven safe and effective pesticides including biological outdoor larvicidal fumigation strategies of water storage containers and public dustings, adequate waste disposal and management, mobilization in environment management and modification underscore the potential health and socio-economic impacts [1, 3, 6, 8].

Third, collective and participative cross sectorial engagement in building active preparedness and response capacity in emergency threats and epidemics require careful and programmatic strategic based on vulnerable population-based programs implementation. Hence, “One Health” approach integration into national IVM is crucial in providing insights into health-animal-environment interface. This can provide early and timely added values for consolidated and harmonized mitigation and adaptation tactics and advocacy in local settings epidemics resurgence. In such leveraging on integrated land resources and water conservancy, agriculture and forestry projects is needed to ensure the implementation of control and elimination approaches in practice. Improvements in islands rural water supply programs, efficient drainage and irrigation modification systems and lakes or rivers engineering in endemic-prone settings should be strengthened coupled with surveillance, health education and research activities.

Fourth, increasing dengue mass literacy and awareness campaigns, intensifying community involvement and empowerment in preventive and control activities, effective risk information communication in improving community perceptive knowledge, dengue resilient attitude and behaviour changes [3, 10, 11]. The quality of emergency care support systems and preventable deaths through integrated programs, adherence to safety standards measures and best practices against emergence and spread *Aedes aegypti* and *A. albopictus* vector resistance to insecticides in communities require further research in most communities endemic areas [13–15]. Taking the advantages of technological advancements such as social media, dengue mass campaign to improve knowledge of disease root-causes, preparedness, prevention and control should be widely spread in promoting dengue resilience culture, capacity and cultural behaviour changes in understanding the ground implementation and operations.

Fifth, improving timely and efficient early alert, social mobilization in increasing community engagement and resilience, health education, awareness campaigns and technical support assistance are needed in robust decision-making in emerging dengue threat and emergency outbreaks response actions [1, 6, 11]. Naturally, strengthening local national and regional “One Health” approach joint policies and activities implementation in enhancing participatory and sustained integrated dengue control into elimination in achieving the global sustainable development goals (SDGs) in attaining national and global health security is crucial.

Sixth, while awaiting safe and effective  dengue vaccines for vulnerable population immunization and travel medicine integration of sensitive and effective laboratories and epidemiological surveillance-response is vital in reinforcing surveillance and control capacity. There is a need to continuously test and monitor *Ae. aegypti* and *Ae. albopictus* sensitive to pesticides, novel rapid early detection tools development and validation for asymptomatic reservoir and active case finding screening and confirmation that will enhance detection, and timely management [5, 7, 11].

Ultimately, the added value of integration of digital IVM-KPIs reporting and laboratory information management system is only crucial in sustained vector(s) prevention and control, but also in ensuring early warning alert timeliness and knowledge-driven programs and interventions effectiveness and coverage. Strengthening health systems in remote rural and urban communities should be established to including adequate housing and precautions guidelines [4, 5, 7]. Gathering of quality dengue and other vector data and database for real-time surveillance and contextual information response and management is essential in forecasting potential dengue resurgence and epidemics. While optimistic and convinced that dengue and related emerging epidemics such as Zika, influenza, malaria are preventable, proactive and collaborative intersectorial and multidisciplinary cooperation amongst all stakeholders including policy-makers, health, agriculture, veterinary, climatic and meteorological, ecology and environmental sectors, Non-governmental organizations and communities commitment and funding in novel approaches development and implementation mainly safe and effective vaccines for mass population immunization is imperative [3, 4, 7, 11, 12, 16, 17].

## Conclusions

There is an urgent call for more integrated operational research on dengue vector-virus-disease behavior and human-animal-environment interactions, and addressing knowledge gaps and challenges in translation into innovative dengue and other arboviral diseases initiatives. Investing in operational dengue diagnostics and laboratory-based surveillance is vital in understanding and mapping contextual determinants responsible for triggering DF and DHF public health threats and epidemics resurgence. Hence, rapid development of more sensitive early dengue detection, diagnostics and surveillance threats and epidemics signals and metrics, safe and cost-effective therapies and dengue vaccines are paramount for vulnerable mass population immunization benefits and well-being in risky-prone settings and travel medicine. As well fostering sustained dengue community-based (school and faith-based approach) cost-effective programs ownership and resilience, health education and awareness campaigns opportunities for health promotion, social responsibility and risk reduction are vital in strengthening health systems capacity in dengue and other emerging or reemerging VBDs (e.g., Zika) sustained control and elimination is advocated.

## Additional file

Additional file 1: Multilingual abstracts in the six official working languages of the United Nations. (PDF 295 kb)
