# Supplementary material for: Outwitting dengue threat and epidemics resurgence in Asia-Pacific countries: strengthening integrated dengue surveillance, monitoring and response systems
Source: Infect Dis Poverty. 2016 May 27;5:56. doi: 10.1186/s40249-016-0148-3 (PMC4884387; doi:10.1186/s40249-016-0148-3)
Supplement: Additional file 1: — Multilingual abstracts in the six official working languages of the United Nations. (PDF 295 kb) [file 40249_2016_148_MOESM1_ESM.pdf]

Translation of the abstract into the six official working languages of the United Nations

تفوق مخاطر حمى الضنك وانتشار جديد للأوبئة فى بلدان آسيا والمحيط الهادئ: تعزيز مراقبة حمى الضنك المتكاملة، وأنظمة الرصد والإستجابة

إرنست تامبو، جون-هو تشن، شياو نونغ تشو، عماد إم خاطر

الملخص

حمى الضنك لا تزال تشكل تهديدا كبيرا كمرض فيروسى تحمله النواقل وعبء على أهمية الصحة العامة فى جميع أنحاء العالم. يعد هذا الوضع صعب للغاية بسبب عودة ظهور فيروس الضنك بطريقة لم يسبق لها مثيل، واستمرار الأنماط المصلية المتنوعة في المناطق المعرضة للوباء وعواقب الأنشطة الطبيعية التى هى من صنع الإنسان والتى بدورها تعمل على تعزيز صحو الناقلات، وآليات انتقال الأمراض وسرعة انتشارها في جميع أنحاء منطقة آسيا والمحيط الهادئ. هناك حاجة ملحة لتعزيز بحث مراقبة الإستجابة فيما يتعلق بالتنفيذ ومحتوى السياق لتحسين الكشف المبكر للكشف عن المستودع النشط، وعقار جديد فى قضية التحقيق والاستجابة القائمة على الأدلة السريعة بما في ذلك نشر تطعيم الضنك الشامل.

علاوة على ذلك، إن مَوْضَعَةٌ الإصابة ومراقبة عوامل خطر حمى الضنك أو محتمات الإصابة، ومشعرات التحكم بالأداء والنتائج أو فعالية برامج المكافحة فى تحديد الحد الأدنى للبيانات الفعلية تجاه قرار المجتمع القائم على المعرفة للسياسة العامة وحزم إستجابة فعالة هو أمر حتمى.

علاوة على ذلك يتعين تنفيذ قوى ومراقبة للإنذار المبكر لحمى الضنك المتكاملة، ومطلوب أيضا قياسات نظم الرصد والإستجابة للأدلة القائمة، فى الوقت المناسب، واستراتيجيات التخفيف للسياق ذو الفاعلية من حيث التكلفة و التدخلات المبتكرة.

Translated from English version into Arabic by nana_2007129, through

TRANSLATORS
WITHOUT BORDERS

强化登革热管控、监测和响应一体化系统，应对亚太国家登革热威胁与疫情复现

Ernest Tambo, 陈军虎, 周晓农, Emad IM Khater

摘要

登革热是一种虫媒病毒疾病，仍给全球公共卫生造成严重的威胁和负担。由于登革热病毒空前复现以及易发流行区存在多种血清型，加之人为和自然活动因素加速推动病媒出现和传播动力学造成登革热在亚太地区传播，促使登革热疫情复杂化。目前，迫切需要加强具有可操作性和环境监测-响应的研究，以提高对宿主检测的早期诊断准确性，加强个案调查中的新药研究和基于证据的快速应答，包括部署大规模登革热疫苗接种。此外，对登革热危险因素或决定因素，控制或消除规划效果中以确定最小有效数据的过程和结局指标进行持续观测和制图是制定社区循证决策和有效应对策略的必要条件。而且，需要实施一个强有力、一体化的登革热预警监测和响应系统以快速有效的制定循证减灾策略，进行干预措施创新。

Translated from English version into Chinese by Chen Jin, edited by Yang Pin through

CDC CHINESE CENTER FOR DISEASE CONTROL AND PREVENTION
NATIONAL INSTITUTE FOR PARASITIC DISEASES

Vaincre la menace de la dengue et la résurgence de l'épidémie dans les pays de la zone Asie-Pacifique : renforcement des systèmes intégrés de surveillance, de contrôle et de réponse

Ernest Tambo, Jun-Hu Chen, Xiao-Nong Zhou, Emad IM Khater

Résumé

La dengue est une maladie virale à transmission vectorielle qui reste une menace et un fardeau de santé publique considérable dans le monde entier. Cette situation est encore aggravée par une résurgence sans précédent du virus de la dengue, la persistance de différents sérotypes dans les régions susceptibles aux endémies, les activités humaines et phénomènes naturels favorisant l'émergence du vecteur, ainsi que par la dynamique de transmission et par l'expansion de la maladie dans la région Asie-Pacifique. Il est urgent de renforcer la recherche opérationnelle et contextuelle sur la surveillance et la réponse en améliorant la détection précoce des réservoirs actifs, en développant les études de nouveaux médicaments et en apportant une réponse rapide et basée sur des donnée probantes, avec notamment le déploiement d'une vaccination de masse contre la dengue. Une cartographie et une surveillance des facteurs de risque et des déterminants de la dengue sont en outre nécessaires, tout comme des indicateurs de performances et d'efficacité des programmes de lutte et d'élimination, à l'appui de décisions basées sur les connaissances communautaires et de systèmes de réponse efficaces. Enfin, de solides critères d'évaluation des systèmes d'alerte précoce, de surveillance et de réponse rapide doivent être définis afin d'élaborer des stratégies contextuelles de lutte contre l'épidémie basées sur les preuves, rapides et efficaces et des interventions innovantes.

Translated from English version into French by Suzanne Assenat, through

Как победить угрозу лихорадки денге и новый рост эпидемии в странах Азиатско-Тихоокеанского региона: Усиление интегрированных систем по наблюдению, мониторингу и реагированию на лихорадку денге

Эрнест Тамбо, Джун-Ху Чен, Ксяо-Тонг Жу, Эмад ИМ Кхатер

Краткое описание ситуации

Лихорадка денге, трансмиссивное вирусное заболевание, до сих пор является серьёзной угрозой и проблемой здравоохранения во всём мире. Ситуация осложняется беспрецедентным ростом и устойчивостью различных стереотипов вируса денге в районах с близкой к эндемической ситуацией, последствиями человеческой и природной активности, способствующими возникновению чрезвычайной ситуации, связанной с переносом инфекции, динамике её распространения и охвату всё больших территорий Азиатско-Тихоокеанского региона. Существует острая необходимость усилить операционное и контекстное исследование по наблюдению и реагированию на ситуацию путём улучшения раннего обнаружения активных резервуаров, применения новых лекарств при исследовании случая и способствования немедленного применения научно обоснованного опыта, включая введение массовой вакцинации от лихорадки денге. Кроме того, необходимо постоянное отслеживание на карте и наблюдение за факторами риска лихорадки денге или детерминант, индикаторами осуществления и результатов программ контроля и противодействия, эффективное определение минимально значимой информации для политики принятия решений обществом, основанной на имеющихся знаниях и эффективных блоках

реагирования. Кроме того, применение прочной интегрированной системы по раннему предупреждению лихорадки денге, системы показателей мониторинга и реагирования необходимы для своевременных, экономичных и основанных на научном опыте стратегий предупреждения негативных последствий и для инновативного вмешательства.

Translated from English version into Russian by Tatiana Glazina, through

TRANSLATORS
WITHOUT BORDERS

Cómo superar la amenaza y el resurgimiento de la epidemia del dengue en los países de Asia y el Pacífico: Fortalecimiento de los sistemas integrados de vigilancia, monitoreo y respuesta para el dengue

Ernest Tambo, Jun-Hu Chen, Xiao-Nong Zhou, Emad IM Khater

Resumen

El dengue continúa siendo una amenaza significativa de enfermedad viral transmitida por vector y una importante carga para la salud pública a nivel mundial. La situación se complica por el resurgimiento sin precedentes del virus del dengue y la persistencia de distintos serotipos en las zonas endémicas más proclives, actividades naturales y artificiales que resultan en la promoción de la emergencia de vectores, la dinámica de transmisión y propagación en la región de Asia y el Pacífico. Existe una necesidad inmediata de robustecer las investigaciones operativas y contextuales en cuanto a vigilancia de respuestas para mejorar la detección precoz de detección de depósitos activos, nuevas drogas en la investigación de casos y una pronta respuesta basada en evidencia, incluida la utilización de campañas de vacunación masiva contra el dengue. Más aún, son imperativos el mapeo sostenible y la observación de factores de riesgo o determinantes del dengue, indicadores de desempeño y resultados de control o eliminación, eficacia de los programas para definir la mínima información necesaria para políticas comunitarias de toma de decisión fundamentadas y paquetes de respuesta efectivos. Además, para el dengue se requiere de la implementación de un sistema de medición robusto e integrado para la vigilancia, monitoreo y respuesta con un sistema de alerta temprana, que permita llevar a cabo intervenciones innovadoras y estrategias de mitigación oportunas, económicas y fundamentadas en la evidencia.

Translated from English version into Spanish by Maria Alejandra Aguada, through

TRANSLATORS
WITHOUT BORDERS
